# Self-reported Tinnitus and Vertigo or Dizziness in a Cohort of Adult Long COVID Patients

**DOI:** 10.3389/fneur.2022.884002

**Published:** 2022-04-25

**Authors:** Chantal Vanessa Degen, Marie Mikuteit, Jacqueline Niewolik, Dominik Schröder, Kai Vahldiek, Urs Mücke, Stephanie Heinemann, Frank Müller, Georg Martin Norbert Behrens, Frank Klawonn, Alexandra Dopfer-Jablonka, Sandra Steffens

**Affiliations:** ^1^Department of Otorhinolaryngology, Hannover Medical School, Hanover, Germany; ^2^Dean's Office—Curriculum Development, Hannover Medical School, Hanover, Germany; ^3^Department for Rheumatology and Immunology, Hannover Medical School, Hanover, Germany; ^4^Department of Computer Science, Ostfalia University of Applied Sciences, Wolfenbüttel, Germany; ^5^Department of Pediatric Hematology and Oncology, Hannover Medical School, Hanover, Germany; ^6^Department of General Practice, University Medical Center Göttingen, Göttingen, Germany; ^7^German Center for Infection Research, Brunswick, Germany; ^8^Biostatistics Research Group, Helmholtz Centre for Infection Research, Brunswick, Germany

**Keywords:** Long COVID, SARS-CoV-2, tinnitus, vertigo, dizziness, Germany

## Abstract

Tinnitus, vertigo and dizziness are symptoms commonly reported among Long and Post COVID patients, however the severity of these symptoms has not been assessed in large trials. Therefore, in this study a large cohort of Long COVID patients was surveyed about the presence and severity of tinnitus and vertigo or dizziness symptoms. The online survey was completed by a German cohort of 1,082 adult Long COVID patients after a mean period of 43.2 weeks ± 23.4 weeks after infection. Eighty percent were not fully vaccinated (at least two vaccinations) at the time of their first COVID symptoms and 9.8% were hospitalized in the course of their acute SARS-CoV-2 infection. At the time of the survey, 60% of patients reported the presence of vertigo or dizziness with a mean severity of 4.6 ± 2.7 on a scale of 1 (least severe) to 10 (most severe) and 30% complained of tinnitus with a mean severity of 4.8 ± 3.0. Approximately one fifth of the participants with tinnitus and vertigo or dizziness, rated their symptoms to be severe. The data shown in this study confirms that tinnitus and vertigo or dizziness are common symptoms in Long COVID patients and demonstrates, that a compelling number of patients rate their symptoms as severe. The self-reported severity highlights the need for Long COVID clinics to address these symptoms effectively. We suggest a multidisciplinary diagnostic and therapeutic approach to prevent further morbidity and socioeconomic burden for Long COVID patients suffering from severe vertigo, dizziness or tinnitus.

## Introduction

Despite extensive preventative measures, testing capacity and accessibility to vaccines, millions of people continue to become infected with variants of the coronavirus, SARS-CoV-2. The surge of the omicron variant has led to record highs in the COVID-19 incidence across Germany and many other European countries in 2022. Health care providers are confronted with increasing numbers of patients, who have recovered from the acute stages of the disease but have not regained their previous level of health and wellbeing or deteriorated again after an initial phase of recovery, a condition termed “Long COVID” or “Post COVID”, if the symptoms last longer than 4 weeks (in the following we use the term Long COVID for these patients). The spectrum of Long COVID symptoms is wide (1, 2) and may often include the neurotological symptom complex of tinnitus, hearing loss and vertigo or dizziness (3, 4).

Neurotological impairments are a known complication of several viral infections (5), however the mechanisms behind virus-associated inner ear dysfunction are still disputed and likely to be multifactorial. Jeong et al. demonstrated that the receptors necessary for tissue invasion by SARS-CoV-2 are expressed in the human inner ear (6). This suggests that viral infection could directly cause inner ear damage leading to hearing loss, tinnitus or imbalance and vertigo. Other explanations for COVID-associated inner ear damage include occlusions of the cochlear or vestibular microvasculature, due to the hypercoagulable state often seen in COVID patients (7).

Current data from cohort studies suggest that neurotological symptoms like tinnitus and vertigo or dizziness are more common among Long COVID patients than the general population, with prevalence estimates ranging between 16 and 26% for tinnitus, and 37 and 43% for vertigo and/or dizziness (1, 8, 9). However, the presence of tinnitus, vertigo or dizziness as a symptom does not sufficiently reflect the morbidity incurred by these patients, nor the need for counseling and treatment. For this, a measure of symptom severity is required. Reports of neurotological symptoms during active SARS-CoV-2 infection have shown variable degrees of hearing impairment, tinnitus, and vertigo or dizziness, ranging from unnoticeable but measurable, to severe and disabling (3, 10). The severity of neurotological symptoms in Long COVID patients has not been documented up to this point.

Therefore, in this study, we sought to determine not only the prevalence, but also the self-reported severity of tinnitus and vertigo or dizziness in a large Long COVID cohort and the potential clinical and socioeconomic significance.

## Methods

A survey was created by a multicenter interdisciplinary team investigating health and social effects of COVID-19. Online access to the questionnaire was provided to minimize potential barriers to participation. The digital questionnaire could be accessed through a link on the project website (www.defeat-corona.de), or by scanning a QR code, that was displayed on posters and flyers at participating medical and research centers. In addition, study information was sent to 400 randomly-chosen primary care, internal medicine, radiology and occupational therapy practices across the region of Lower Saxony, Germany. The link was shared in online patient advocacy groups and web pages of the participating institutions. The survey was conducted in plain German language using the SoSci Survey online platform (www.soscisurvey.de) and consent was given digitally by all respondents. The collected data contained no personally identifiable information. Pseudonyms were assigned to participants open to taking part in follow-up research. Data from participants who were at least 18 years old with symptoms more than 4 weeks after a SARS-CoV-2 infection, confirmed by a self-reported positive PCR, antibody or antigen test, were included in the study. Participants rated the severity of each symptom on a scale of 0 to 10, 0 representing the absence of that symptom and 10 being the most severe level of the symptom [“What symptoms have you experienced since COVID (first appeared during or after disease)? – Tinnitus | Vertigo /dizziness”]. A score of 8 or higher was regarded as very severe. We pooled the symptom of vertigo and dizziness into one item, because most patients do not differentiate between these symptoms.

Participants with at least two vaccinations before the onset of COVID-19 symptoms were regarded as fully vaccinated. To adjust for possible concentration difficulties due to persistent COVID symptoms, it was possible to interrupt the questionnaire and continue at a different point in time. The implementation and data protection strategies were assessed and approved by the ethics committees of the participating institutions. The study was registered in the German register for clinical trials (DRKS00026007). The survey opened in September 2021 and is still ongoing.

The table displaying demographic data of the survey participants shows the mean and standard deviation of numerical variables like age and the time from infection to survey. Categorical variables are presented in number of participants and proportion of the survey population. The number of participants for each value on the symptom scale of tinnitus and dizziness or vertigo is shown. Additionally, the symptom intensity mean and standard deviation of symptomatic participants is presented. Participants without symptoms were excluded from the calculation of the mean and standard deviation.

## Results

### Demographic Data

For this study data was collected between September 2021 and January 2022. The questionnaire was completed by 1,082 patients complaining of symptoms at least 4 weeks after SARS-CoV-2 infection. In 94% of patients, diagnosis was confirmed by a PCR test. Participants were mostly female (72.9%). 9.8% of all participants required hospitalization during their acute infection, with 2.4% needing intensive care treatment. Fifteen percent of patients were fully vaccinated prior to infection. The age distribution and other demographic data is shown in Table 1.

**Table 1:** Demographics of survey respondents with Long COVID.

**Variable/Data**	**Long COVID**	
Number of participants (n)	1,082	
Time from beginning of the infection to survey (weeks)	43.2 (23.4)	
**Gender n (%)**		
Female	789 (72.9)	
Male	208 (19.2)	
Diverse	3 (0.3)	
Not answered	82 (7.6)	
Age Group (years) n (%) | mean (sd)	1,082 (100)	43.0 (12.4)
18–29	170 (15.7)	24.8 (3.1)
30–39	223 (20.6)	34.6 (3.0)
40–49	261 (24.1)	44.4 (3.0)
50–59	260 (24.0)	54 (2.8)
60–69	76 (7.0)	62.8 (2.7)
70+	10 (0.9)	73.7 (5.4)
Not answered	82 (7.6)	—
**Hospitalization n (%)**		
No	966 (89.3)	
Yes, regular ward	80 (7.4)	
Yes, intensive care unit	26 (2.4)	
Unknown / not answered	10 (0.9)	
Sars-CoV-2 Test^*^n (%)		
PCR Test positive	1,021 (94.4)	
Anti-Body Test positive	143 (13.2)	
Antigen Rapid Test positive	43 (4.0)	
**Vaccination status against Sars-CoV-2 n (%)**		
Yes (overall)	915 (84.6)	
Yes (prior infection)	167 (15.4)	
No	100 (9.2)	
Unknown / not answered	67 (6.2)	

* *Multiple selection possible*.

### Dizziness, Vertigo and Tinnitus in Long COVID Patients

In this cohort of 1,082 Long COVID patients, neurotological symptoms were common, with 60% suffering from some degree of vertigo or dizziness and 30% reporting tinnitus (Table 2). Two hundred sixty six (24.6%) participants reported suffering both from tinnitus and vertigo or dizziness and 275 (25.4%) participants showed neither of the symptoms. The survey was taken an average of 43.2 weeks ± 23.4 weeks after the first symptoms of the SARS-CoV-2 infection. The self-reported severity of symptoms was assessed with a scale of 1 to 10 (0 represented the absence of the symptom). Table 3 shows the results of the self-assessment of symptom severity for tinnitus as well as dizziness or vertigo. Three hundred thirty two patients reported tinnitus with a mean severity of 4.8 and a standard deviation of 3.0. Milder symptom severity was more common for tinnitus, but 23% of patients with tinnitus rated their symptoms as very severe (8 or higher). Albeit twice as prevalent in this cohort compared to tinnitus, dizziness and vertigo scores showed a similar severity distribution. The mean self-reported severity for vertigo or dizziness was 4.6 ± 2.7 and 20% of patients with vertigo or dizziness assessed their symptom severity as 8 or higher on the scale from 1 to 10 (Table 3).

**Table 2:** Self-reported symptoms of survey respondents with Long COVID.

**Symptoms**	**Long COVID *n* = mentions (%)**
Tinnitus	332 (30.6)
Dizziness or Vertigo	647 (59.8)
Tinnitus & Dizziness or Vertigo	266 (24.6)

**Table 3:** Self-reported severity of symptoms on a scale of 0 (no symptoms) to 10 (most severe symptoms).

	**0** **=** **no symptoms**							**10** **=** **most severe symptoms**					
	**0**	**1**	**2**	**3**	**4**	**5**	**6**	**7**	**8**	**9**	**10**	**mean ±sd**	**not answered**
Tinnitus, n (%)	636 (58.8)	65 (6.0)	33 (3.1)	42 (3.9)	28 (2.7)	28 (2.7)	25 (2.3)	32 (3.0)	36 (3.3)	16 (1.5)	27 (2.5)	4.8 ± 3.0	114 (10.5)
Dizziness or Vertigo, n (%)	339 (31.3)	81 (7.5)	102 (9.4)	77 (7.1)	71 (6.6)	77 (7.1)	49 (4.5)	62 (5.7)	62 (5.7)	29 (2.7)	37 (3.4)	4.6 ± 2.7	96 (8.9)

## Discussion

To further elucidate the frequency and severity of persistent neurotological symptoms after a SARS-CoV-2 infection, a low-threshold digital survey about tinnitus and vertigo or dizziness was conducted on a large German cohort. The results showed that tinnitus and vertigo or dizziness are very common symptoms among Long COVID patients. The severity of symptoms varied, ranging from mild to very severe. Due to a lack of follow-up, it is unclear from our data, whether the severity of these symptoms declines or remains constant over time. In a large survey of 5,163 COVID survivors, Lambert and colleagues questioned participants about 101 distinct symptoms and showed that 37.6% of Long COVID patients reported dizziness and 16.7% complained of tinnitus for a mean duration of 80 and 93 days, respectively (1). Thrane et al. reported follow-up data on 21 COVID patients with tinnitus, 7 of whom had recovered after 259 days (9). However, a large cohort study by Davis et al. showed an increase of tinnitus prevalence in Long COVID patients after infection from 11.5% (CI 10.47–12.52) at week 1 to 26.10% (CI 23.51–29.10) at the final follow up after seven months (8). These numbers are consistent with the tinnitus prevalence recorded in our Long COVID cohort (30% after an average of 43 weeks). On the other hand, the proportion of patients with dizziness, vertigo, unsteadiness or other balance issues was considerably higher in our cohort (60%), compared to the study by Davis and colleagues (27% at 7 months; CI 24.28–29.81). The reason for this difference may be due to the sensitivity of our survey, which was able to distinguish even very mild forms of dizziness or tinnitus. The dissemination of the survey in online patient advocacy groups may have caused a selection bias toward patients more strongly affected by Long COVID symptoms.

Based on the age distribution of our cohort, the prevalence of self-reported tinnitus and dizziness or vertigo symptoms was higher compared to similar populations without Long COVID. Estimates on tinnitus prevalence vary widely, especially depending on the age of the studied population and what time frame the question is referring to (11). In a cross-sectional study surveying a representative sample of more than 3000 German citizens, Pilgramm et al. determined the prevalence of chronic tinnitus among German adults to be 3,5% (12) and reported that half of the survey participants with chronic tinnitus did not perceive it as bothersome. A similar cross-sectional study in the US found that 7.9% of US adults experience tinnitus at least once a day (13). In a survey of 2064 randomly sampled primary care patients in London, 23% of working adults reported that they had experienced dizziness or vertigo within the previous month (14).

Even though only about one in five patients with tinnitus, vertigo or dizziness assessed their symptoms as subjectively very severe, the increasing number of COVID-19 raises concern about the rising number of patients experiencing these debilitating sequelae. Within the last 2 years more than 20 million Germans have been infected with SARS-CoV-2. It is unclear how long these symptoms persist after the infection, but based on this survey, at least a transient pandemic-related increase in the population prevalence of tinnitus and vertigo or dizziness is to be expected.

The pathophysiology behind these symptoms is yet to be established. Especially when pooling rotational vertigo with unsteadiness, dizziness and imbalance, the underlying mechanisms are likely to be multifactorial. Tinnitus is now thought to be a complex neurophysiological interplay between auditory and non-auditory systems (15). The emotional distress caused by prolonged morbidity after a COVID-19 may be a contributing factor to the increasing prevalence of tinnitus in Long COVID patients over time. The odds of having frequent tinnitus were shown to be strongly increased in people with anxiety (13) and depression was reported to be a stronger predictor for tinnitus severity than hearing loss (16). Anxiety and emotional upset over persistent symptoms, as well as the sociocultural and socioeconomic effects of the pandemic could therefore be associated with tinnitus prevalence and severity in this cohort. In an international cohort of individuals with pre-existing tinnitus, 31% reported more bothersome tinnitus-symptoms during the pandemic due to lifestyle changes, health concerns and social distancing (17). Also, the emotional state during the pandemic played a role in the self-assessment of pre-existing tinnitus symptoms.

Overall, the symptoms of acute COVID-19 and its sequelae are highly variable. Yet, this study identified two common neurotological symptoms among Long COVID patients, that can severely affect quality of life. Whether objectifiable or not, patient reported outcome measures are of particular relevance in the study of Long COVID, to provide a sensible estimate of what share of the adult Long COVID patients experience severe symptoms and may therefore seek consultations or treatment for their neurotological symptoms. This information is essential for health care providers adjusting their services to the demands of patients suffering from post COVID-19 sequelae. The high prevalence of neurotological symptoms like vertigo, dizziness and tinnitus highlights the need for interdisciplinary treatment approaches in Long COVID clinics involving specialists for Otorhinolaryngology and psychosomatic disorders. From a public health standpoint, it is important to gauge the prevalence and severity of these symptoms and to make appropriate resources available. Participants in this study reported on the severity of their symptoms on an arbitrary scale of 1–10. It is unclear whether there is a discrepancy between the severity of the symptoms, for example perceived loudness of tinnitus, and the impact on quality of life in this cohort. Patients may differ significantly in their ability to cope with symptoms, and symptom severity may not be a strong predictor for seeking treatment. In addition, the differentiation between dizziness and vertigo is difficult for patients to self-assess. These weaknesses could have been avoided using detailed validated questionnaires like the tinnitus functional index (TFI) or European Evaluation of Vertigo scale (EEV). However, we know that up to one in three Long COVID patients struggle with impaired cognitive function, often described as “brain fog” (18). Using the 25-item TFI or similar questionnaires would likely have caused a selection bias toward patients without neurocognitive impairments and reduced overall turnout in this online survey format.

Further research is needed to determine whether receiving vaccinations before the infection affects the likelihood of developing Long COVID symptoms. Furthermore, it is yet to be determined how the SARS-CoV-2 variants differ with regard to the Long COVID symptom spectrum. This research is especially difficult to perform, as sequencing capacity is still limited, and most patients do not receive information on the variant they are infected with.

In conclusion, in this study of Long COVID patients, neurotological symptoms were common, with 60% suffering from some degree of vertigo or dizziness and 30% reporting tinnitus. Both symptoms have a multifactorial etiology and require multidisciplinary cooperation for adequate diagnostics and treatment. Therefore, it is important that Long COVID clinics develop multidisciplinary diagnostic and therapeutic concepts for Long COVID patients with tinnitus, dizziness and vertigo. Further morbidity and socioeconomic burden resulting from this pandemic can only be prevented, if appropriate care is provided to Long COVID patients.

## Data Availability Statement

The raw data supporting the conclusions of this article will be made available by the authors, without undue reservation.

## Ethics Statement

The studies involving human participants were reviewed and approved by the Ethics Committee of Hannover Medical School (Approval No. 9948 BO K 2021) and registered in the German Clinical Trial Registry (DRKS00023972). Informed written consent was obtained from all individual participants included in the study. Details that disclose the identity of the subjects under study were omitted.

## Author Contributions

CD, MM, and SS participated in the data interpretation and drafting of the manuscript. KV and DS performed the statistical analysis. JN and MM greatly contributed to data acquisition. AD-J and SS coordinated the project. All authors contributed to data interpretation, revised the manuscript for important intellectual content, and read and approved the final manuscript.

## Funding

This study was part of the DEFEAT Corona project, funded by the European Regional Development Fund (EFRE, Funding No: ZW7-85152953). The funding source had no influence on the design or execution of the study, data analysis or interpretation.

## Conflict of Interest

The authors declare that the research was conducted in the absence of any commercial or financial relationships that could be construed as a potential conflict of interest.

## Publisher's Note

All claims expressed in this article are solely those of the authors and do not necessarily represent those of their affiliated organizations, or those of the publisher, the editors and the reviewers. Any product that may be evaluated in this article, or claim that may be made by its manufacturer, is not guaranteed or endorsed by the publisher.
